# A QoE-Oriented Uplink Allocation for Multi-UAV Video Streaming

**DOI:** 10.3390/s19153394

**Published:** 2019-08-02

**Authors:** Chao He, Zhidong Xie, Chang Tian

**Affiliations:** 1 College of Communications Engineering, Army Engineering University of PLA, Nanjing 210007, China; 2 National Innovation Institute of Defense Technology, Academy of Military Sciences of PLA, Beijing 100071, China

**Keywords:** UAV cluster, uplink allocation, QoE, potential game, distributed self-learning algorithm, H.265

## Abstract

Video streaming has become a kind of main information carried by Unmanned Aerial Vehicles (UAVs). Unlike single transmission, when a cluster of UAVs execute the real-time video shooting and uploading mission, the insufficiency of wireless channel resources will lead to bandwidth competition among them and the competition will bring bad watching experience to the audience. Therefore, how to allocate uplink bandwidth reasonably in the cluster has become a crucial problem. In this paper, an intelligent and distributed allocation mechanism is designed for improving users’ video viewing satisfication. Each UAV in a cluster can independently adjust and select its video encoding rate so as to achieve flexible uplink allocation. This choice relies neither on the existence of the central node, nor on the large amount of information interaction between UAVs. Firstly, in order to distinguish video service from ordinary data, a utility function for the overall Quality of Experience (QoE) is proposed. Then, a potential game model is built around the problem. By a distributed self-learning algorithm with low complexity, all UAVs can iteratively update their own bandwidth strategy in a short time until equilibria, thus achieving the total quality optimization of all videos. Numeric simulation results indicate, after a few iterations, that the algorithm converges to a set of correlation equilibria. This mechanism not only solves the uplink allocation problem of video streaming in UAV cluster, but also guarantees the wireless resource providers in distinguishing and ensuring network service quality.

## 1. Introduction

The great progress in aviation, new energy and artificial intelligence (AI) technologies has led to the rapid development of unmanned aerial vehicles (UAVs). They are becoming much smaller, lighter and more intelligent and are widely used in both civilian and military fields. UAVs can acquire images and videos in real time by the video sensors they carry. Meanwhile, they compress and encode the multimedia, and finally upload them through the wireless network. This way of real-time shooting and transmission makes UAVs play a great role in civil fields, such as land mapping, pollution monitoring, disaster management, and personal aerial photography [1,2,3]. In terms of military applications, UAVs have penetrated into all aspects of combat. The panoramic real-time video brought by military UAVs overturns the traditional close-in reconnaissance, situational awareness, fire evaluation and other military operation modes. The clusters of multi-UAVs, working cooperatively, are of great significance for the army to obtain battlefield information right.

Although UAV videos have brought great convenience, they are a kind of information that may occupy a lot of resources, and the transmission capacity of wireless channel is limited. Current video compression algorithms have been developed to be quite mature, such as H.264/AVC and H.265/HEVC, which can remove the redundancy in video signals more effectively. However, as the video resolution has been greatly improved, the requirement of wireless resource will also drastically increase.

The coverage, information acquisition ability and destruction resistance of a single UAV is relatively limited. These problems can be solved well in the UAV cluster. A cluster of UAVs can present information on a much larger scale and from more angles [4]. However, in addition to the convenience, the UAV cluster has many other issues which need to be further considered, such as the communication links, the networking mode, routing mode, etc. Especially when their flying area is relatively concentrated, the shared wireless resources over a small area will not be infinite, which means they have to compete with each other. As a result, the effective allocation of resources will become very necessary.

The insufficiency of channel resource may deteriorate the quality of video transmission, so we mainly consider the situation of limited channel bandwidth in the paper. The question of how to measure the deterioration, or from another aspect, how to measure the utilities, should be answered first. We need an objective metric. As an application layer service, the ultimate goal of transmitting video is to provide users with better viewing experience. Therefore, Quality of Experience (QoE) is a better satisfaction metric. The total QoE of all the videos could provide a clear indicator to evaluate the allocation effects. In this situation, the transmission rate is the main factor that could influence the QoE value. Therefore, when multi-UAVs in a cluster separately uplink videos through a wireless access point at the same time, how to allocate the limited bandwidth resource among them in order to maximize the total QoE has become an urgent and complex problem. The main contributions of this paper are summarized as follows:

- We study the issue of rate allocation when multi-UAVs capture videos and send them back via the wireless channel simultaneously. The total QoE of all videos are considered as the optimization goal and the costs for channel renting and energy consumption have been deducted.
- Based on the potential game, we build a new distributed resource allocation framework. According to the potential function we propose, the game is proved as a complete potential game and the correlated equilibrium of the game exists and is unique.
- In order to make all the UAVs in the cluster iteratively update their bandwidth strategy, we adopt a distributed self-learning algorithm, by which the correlated equilibrium could be achieved with a relatively fast convergence rate.
- The rate ranges and characteristics of some real videos are analysed and these videos are applied in the simulations. From the real-time flow rate and total utility, we find that the algorithm converges rapidly and each UAV can intelligently select and maintain a stable video uplink rate, so that a reasonable allocation of wireless resources could be achieved. The influence of total channel bandwidth and cost factor is also analyzed.

The rest of this paper is organized as follows. In Section 2, the related works are summarized. In Section 3, we describe the system model in detail and discuss our preliminary goal. In addition, the utility function is introduced here. In Section 4, we model the problem as a potential game and prove the existence of correlated equilibrium. We adopt a distributed self-learning algorithm to solve the model in Section 5. The experimental results on real video data and some discussions are settled in Section 6. In addition, we draw the conclusions in Section 7.

## 2. Related Works

There are many related studies on UAV resource allocation. Usually, they are different from three aspects: the goal, the object, and the method. Different allocation goals will bring totally different results. Unlike some methods for improving the physical layer parameters, the resource allocation for video transmission should naturally be set from the application layer. The object determines the main body of the allocation. At this point, the UAVs flying in cluster are not only different from single UAV, but also different from other mobile networks. Allocation methods are the concrete algorithms and steps adopted in the process. Because of the particularity of UAV cluster, a decentralized method may guarantee more robustness of the system.

### 2.1. The Allocation Goal

QoE is defined as the degree of delight or annoyance of the user of an application or service [5]. Compared with Quality of Service (QoS), which is limited to the measurement of objective parameters, QoE covers domains beyond telecommunication. The factors of the system, human, context and content can all influence the QoE results a lot [6]. Thus, it is very important for network providers, service providers, device manufacturers and end users.

When delivering multimedia, QoE will be affected by more specific issues, such as video capture, coding, storage, delivery, decoding, rendering, display, context of use and user factors [7]. These complex factors determine that the effects of transmission cannot be simply attributed to QoS. The true visual experience of individual users need to be represented in a precise and comprehensive way. Furthermore, the changing conditions of UAV networks with fluid topology may also introduce numerous problems in video transmission. Therefore, it is more important to study the consequences from the viewers’ perspective.

Some research in wireless transmission has better analyzed and solved the issue of resource allocation by improving QoE [8,9,10,11]. Thus, resorting to the mature QoE model to allocate network resources has become popular. Meanwhile, the research on UAV is closely related to QoE, too [12,13,14]. In order to make the allocation mechanism serve the UAV video transmission much better, we also regard users’ experience as our goal and foundation of the study.

### 2.2. The Allocation Object

The communication links are different in multi-UAV networks and they can be summarized into four categories: Air-to-Ground (A2G), Ground-to-Air (G2A), Air-to-Air (A2A), and Ground-to-Ground (G2G) [15]. Among them, A2G links may adopt IEEE 802.11 standard or other customized networks, which could provide high speed data transmission and large-scale coverage. It is also the typical data service link for video transmission. The inter-UAV links of A2A may use the low power consumption and less complicated standard such as the IEEE 802.14.4. The network topologies can be classified as star, multi-star, mesh and hierarchical mesh [16].

**Figure 1:**
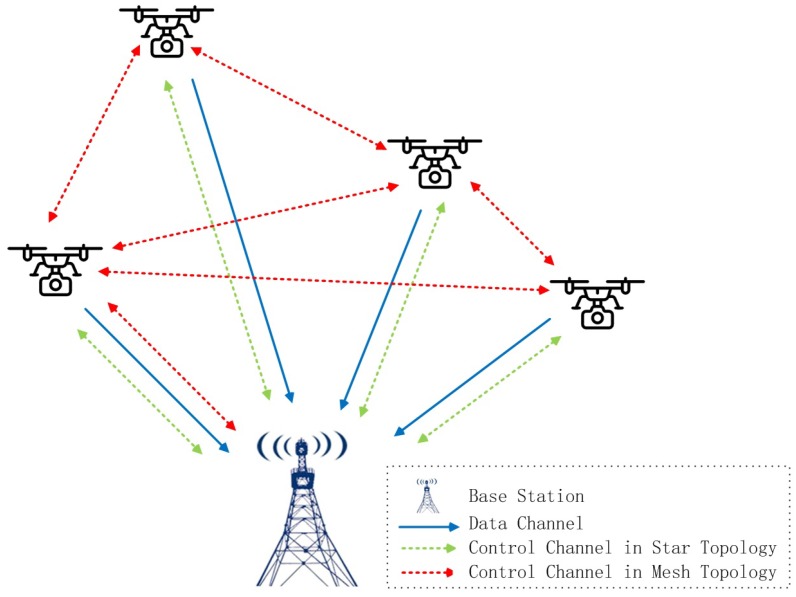
A typical topology for communication of multi-UAVs.

In wireless communication systems, there are usually two kinds of channels: control channel and data channel. A typical UAV cluster communication topology is shown in Figure 1. Due to the relative motion between UAVs, multi-UAVs will constitute a fluid topology, which will lead to the instability of inter-UAV communication. Usually, if UAVs collect a large amount of data to transmit, such as high-resolution video, they mainly rely on A2G links and use the star network topology. The blue straight lines in the figure show the video data link, and the video captured by each UAV is sent directly to the base station.

From the view of control channel, there are usually two kinds of ways for the control information transmission. One is achieved by the star topology, which relies on the central node to uniformly schedule resource allocation, as shown by the green dotted line in Figure 1. In these centralized control algorithms, the base station as the central node should know the parameters of all UAVs, based on which resources can be calculated in a collective way. This method can make full use of the computing advantages of the central node. It has certain advantages in some specific scenarios [17,18,19]. However, in star networks, central nodes need to predict the information of all of the UAVs. It is difficult to be realized in the application service oriented allocation. In addition, all of the information exchanges among UAVs would be through the base station and the occurrence of blockage of links and higher latency would bring adverse effects.

The other way to realize the allocation control is through the distributed information interaction between UAVs in mesh topology, as shown in the red dotted line in Figure 1. Distributed algorithms have the characteristics of high efficiency, scalability and robustness, and have become the mainstream methods of resource allocation. It avoids the complex computation of the central node and, to some extent, saves the network resources. Ref. [20] determined the distributed uplink transmit power of IoT nodes. Ref. [21] proposed a distributed power allocation algorithm based on an alternating direction method of multipliers in UAV-assisted networks. Ref. [22] took game theory as a distributed way for resource allocation. However, the specific implementation of allocation is different from traditional mobile networks, such as the Ad Hoc Network. The multi-UAV formation is dynamic and the topology of the cluster is fluid. This may bring the possible intermittence of the A2A communication link and lead to the failure of control information transmission.

In addition to the links and networking issues, the problems of flying altitude, trajectory and speed, completion time, energy consumption, etc. [23,24] also need to be considered in multi-UAV communication. All of them may bring challenges to the resource allocation of the UAV cluster.

### 2.3. The Allocation Method

Although the traditional optimization method can better solve the problem of wireless resource allocation, not all problems can easily get the optimal solution. Especially when the optimization object contains multiple elements, the complexity of optimization methods will be further increased. This will bring a huge computing burden and big time delay to the real-time transmission service. In addition, the payloads of small UAVs cannot afford the high complex calculations. As we need to take the cluster of UAVs’ video transmission into consideration, in order to avoid the complexity above, game theory method is chosen as the resource allocation algorithm in this paper. Game Theory is a branch of applied mathematics and the players can interact among themselves to obtain a stable allocation of system resources [25]. In general, it can be broadly divided into cooperative and non-cooperative games. We can find that game theory is already widely used in resource allocation and has played an effective role.

In a cooperative game, the participants reach an agreement to obtain better overall benefits. Cooperative game can be divided into two-person cooperation and multi-person cooperation. The former is often called Nash bargaining problem and the latter is also called coalition game. Ref. [26] modeled the bandwidth allocation problem as a cooperative negotiation and considered both the fairness and the QoE. Ref. [27] regarded the UAVs as flying base stations and took the model of a coalition formation game to deal with the trade-off between coverage and transmission performance. Although in the coalition game the multi-players’ benefits are considered comprehensively, in practice, the formation of the coalition is often accompanied by losses, and it is difficult to always form a big coalition. In the optimization process of a coalition formation game, the participants will be selected, which means that some players will be excluded. Thus, a cooperative game method is not applicable to the scenario where each UAV has irreplaceable tasks.

Compared with cooperative games, non-cooperative games pay more attention to the maximization of each player’s own benefit, and their application is more popular. Ref. [28] modeled the offloading task as a replicator dynamic process. Ref. [29] proposed a Stackelberg dynamic game model to get the optimal allocated resources. Ref. [30] uses a non-cooperative game to achieve power allocation scheme. Although the development of non-cooperative game is relatively mature, in reality, the rules of non-cooperation are not absolute. More than that, with the further complexity of communication and network, the single non-cooperative game mode cannot solve the problem of resource allocation well. Ref. [31] established a two-level game framework, with an evolutionary game for underlying service and a differential game for upper bandwidth selection. Ref. [32] solved a spectrum pricing and allocation problem. The hierarchical two-level game framework included a Stackelberg game and a Bargaining game. Ref. [22] proposed a hierarchical game framework to solve the problem of joint access selection and bandwidth allocation. These multi-tier games are often used in scenarios where multiple problems need to be solved comprehensively, for example, when power control and channel selection are considered simultaneously, or when network service providers, secondary suppliers, and users make resource selection at the same time.

Although the studies above have made some achievements, they are improper for the multi-UAV video transmission service. Especially in the application scenario of this paper, UAVs not only need to compete for resources, but also need to consider the overall consequences of video uplink brought by such competition. The nature quality of Potential Game (PG): the motivation of all players to change their strategies can be expressed as a global function [33], which could exactly meet this need. Some studies have applied the potential game to solve the spatial spectrum access problem [34] and power allocation problem [35]. We can also find this game method widely used in resource allocation, especially cooperating with other methods in some complex situations. Ref. [36] modeled the inter-cell channel allocation as a potential game and the intra-cell one as a many-to-one matching game. Ref. [37] solved the bitrate adjustment problem by the large deviation principle and the spectrum allocation problem by a potential game. Although the above two studies incorporated and solved two issues together, they may tend to neglect some details while achieving overall targets and we can find that neither of them considered the characteristics of specific videos. Thus, that’s what we need to do. In this paper, we will discuss the adoption of potential game in rate allocation, especially from the perspective of application layer videos’ particularity and their transmission quality assurance.

In a word, the rate allocation in the UAV cluster for video transmission is not a simple algorithm for resource divisions. The mechanism should consider to improve the video QoE, take the flying cluster as the object, and adopt a distributed method like game theory. In previous related works, these three aspects have not been fully studied and we will discuss about them further in this paper.

## 3. System Model and Utility Function

### 3.1. System Model

**Figure 2:**
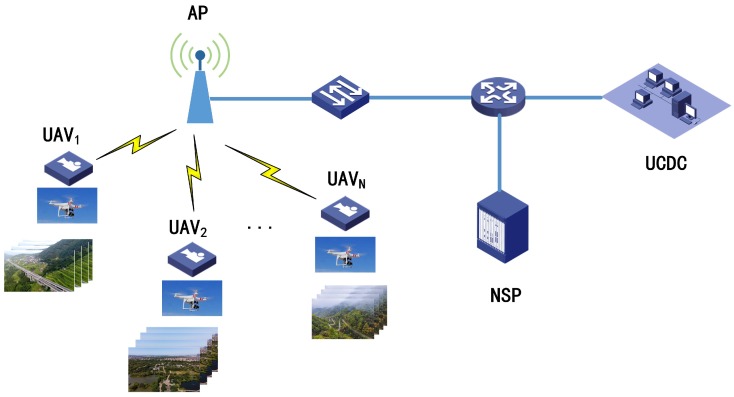
Video uploading of a UAV cluster via a wireless network.

The development of various wireless technologies has greatly expanded the application of UAVs. For example, the Massive Multiple-Input Multiple-Output (MIMO) technology used in 5G network breaks the limitations of traditional 2D-MIMO and increases the vertical dimension, which is more suitable for the three-dimensional nature of UAVs. At present, people have completed the UAV flight communication tests based on the 5G network for many times and achieved High-Definition (HD) live videos’ broadcasting. Not only 5G cellular networks, but also many other wireless technologies could provide UAVs with favorable communication environments [38]. Without loss of generality, in this paper, we suppose several UAVs, as a cluster, are flying in the area covered by a customized network. They perform the tasks of acquiring and uploading videos. Figure 2 shows a typical scenario that needs to be discussed in this paper. There are N UAVs in the network. Suppose they are all in the same coverage area of a wireless Access Point (AP), which belongs to a certain Network Service Provider (NSP).

Each UAV is equipped with video sensors and communication payload for filming and sending the encoded video back via the wireless network. In this process, the flying position and speed of the UAV may be different, and the shooting angle and object may also be different, which makes them get different videos. From the perspective of intra-frame difference, if the background of the image is messy or the targets are numerous, then such video is relatively complex in terms of content. From the perspective of inter-frame difference, if the motion rate of subjects is fast or the change range is large, the video content will be more complex. Video scene fragments collected by different UAVs in the cluster are compressed, encoded and finally sent to the UAV Control and Data Center (UCDC). Besides controlling the flight speed and trajectory of the UAV cluster, the UCDC also integrates and edits the encoded fragments obtained by the UAVs, which are eventually used for various commercial purposes. Meanwhile, UCDC needs to lease wireless channels from the NSP for each UAV. Flight control information usually consumes very few resources, so most of the rental fee is related to the actual channel bandwidth occupied by each video stream. It is hoped that UCDC can obtain high-quality video signals, so that they can meet the users’ needs in QoE finally. When the wireless resource is insufficient, the unfairness of uplink channel allocation will lead to severe transmission error or packet loss of some individuals and eventually affect the total watching experience of all gathered videos. Therefore, the limited bandwidth resources need to be reasonably allocated.

Suppose that Cband represents the total throughput that the AP can provide. Let $r_{i}\in Cband$ represent the channel bandwidth occupied by the video of the *i*-th UAV, which varies from the minimum rate constraint $R_{i}^{\min}$ to the maximum one $R_{i}^{\max}$, i=1,2,…,N. $R=[r_{1},r_{2},\ldots ,r_{N}]$ represents the transmission rate vector of N videos, and $U=[u_{1},u_{2},\ldots ,u_{N}]$ represents the corresponding utility function vector. In order to return high-quality video, each UAV intends to magnify their utilities:

(1) $$\begin{array}{cc} \max\; & u_{i},\\ s.t.\; & R_{i}^{\min}\leq r_{i}\leq R_{i}^{\max},\\ & 0\leq \sum_{i=1}^{N}r_{i}\leq Cband. \end{array}$$

### 3.2. QoE-Based Utility

As mentioned in Section 2.1, we need to implement resource allocation with the goal of improving QoE. Video QoE metrics can also be called Video Quality Assessment (VQA), which can be divided into subjective assessment and objective assessment [39]. Subjective assessment is mainly conducted by the evaluator in a specific test environment, and the Mean Opinion Score (MOS) is obtained. The method and procedure is time-consuming and expensive, which leads people to predict the video quality through the other way. The objective VQA models based on the error statistics of pixel domain are relatively simple, including PSNR, SSIM, MOVIE, etc. [40,41,42]. The models oriented to video features are more complex and more accurate, for example, VQMTQ and Q-STAR [43,44]. It is hoped that, by means of establishing mathematical models, objective assessment could make the predicted results much more approximate to the real MOS value.

On one hand, the video feature-oriented models extract the feature information from the video itself. This is consistent with our method because the analysis in this paper is based on rate ranges and characteristic of each video. On the other hand, the results of the models oriented to video features are closer to real MOS values. Therefore, we start from the model of VQMTQ [43] and further analyze the QoE-oriented utility function. Firstly, from the VQMTQ evaluation method, we can find that the subjective perception quality is closely related to the objective evaluation results PSNR and frame rate, which can be denoted as VQMTQ(PSNR,f)=SQF(PSNR)·TCF(f). In the scenario of this paper, the video captured by UAV needs to be sent to UCDC for clipping, editing and processing together. Thus, the frame rate of all the videos is the same. The above statement is translated into

(2) $$\begin{array}{c} QoE_{MOS}=Const_{fps}\cdot \Psi \left(PSNR\right), \end{array}$$

where $Const_{fps}$ is the constant related to frame rate, and Ψ(·) is the mapping function from PSNR to MOS.

Some studies suggest that there is a simple linear mapping between PSNR and MOS [45,46] and it can be formulated as $MOS=\left\{\begin{array}{cc} 4.5,\; & 40<PSNR,\\ \frac{3.5*PSNR}{20}-2.5,\; & 20<PSNR\leq 40,\\ 1,\; & 0<PSNR\leq 20. \end{array}\right.$ However, Ref. [47,48] show that this mapping is much closer to a sigmoid function. Although PSNR is not the most accurate VQA metric, it is most widely used. Much literature directly uses PSNR as the measurement of QoE [49,50]. Meanwhile, it has the lowest complexity [47] and this makes it more convenient to use in real-time services. Thus, we will apply a simple linear mapping between the PSNR and the MOS:

(3) $$\begin{array}{c} QoE_{MOS}=Const_{fps}\cdot \Psi \left(PSNR\right)=Const_{fps}\cdot \left(A\cdot PSNR+B\right). \end{array}$$

In addition, then, we could formulate the utility function for each UAV video:

(4) $$\begin{array}{c} u_{i}=QoE_{MOS}-COST_{i}=Const_{fps}\cdot \left(A\cdot F\left(r_{i}\right)+B\right)-COST_{i}. \end{array}$$

For $F(r_{i})$, we use the expression of PSNR in [51] and $PSNR=F\left(r_{i}\right)=a+b\cdot \sqrt{\frac{r_{i}}{c}}\left(1-\frac{c}{r_{i}}\right)$. The parameters *a*, *b* and *c* are constants and have been discussed in detail in the literature. $COST_{i}$ represents the payment in the process of transmission.

In this paper, two aspects of transmission cost are considered. One is the charge by NSP due to the occupation of channel resources. The charging way is determined by the ratio between the actual video transmission rate and the total uplink channel bandwidth. Thus, $P\left(r_{i}\right)\;=\;\theta \cdot \frac{r_{i}}{Cband}$, and the constant θ is the price factor. For scenarios where the capacity is large enough or the price is not considered, θ can be zero. The other is the cost of energy consumption. For all UAVs, the energy consumption of video capturing and encoding is roughly the same. Thus, we mainly consider the energy loss caused by transmission, which is related to the video encoding rate. $Q\left(r_{i}\right)=\delta \cdot r_{i}$ and the constant δ is the energy factor. The higher the rate, the more the energy consumption of transmission. Therefore, $COST_{i}=P\left(r_{i}\right)+Q\left(r_{i}\right)$ and the utility for each UAV can be formulated as

(5) $$\begin{array}{cc} u_{i} & =QoE_{MOS}-COST_{i}\\ & =Const_{fps}\cdot \left\{A\cdot \left[a+b\cdot \sqrt{\frac{r_{i}}{c}}\left(1-\frac{c}{r_{i}}\right)\right]+B\right\}-\theta \cdot \frac{r_{i}}{Cband}-\delta \cdot r_{i}\\ & =\Omega \cdot \left[a+b\cdot \sqrt{\frac{r_{i}}{c}}\left(1-\frac{c}{r_{i}}\right)\right]-\eta \cdot r_{i}+\Lambda , \end{array}$$

where Ω and Λ are the constant terms after the combination of the above QoE factors, and η is the constant term after the combination of rental fee of the network and energy factors.

## 4. Potential Game Based Uplink Allocation

Compared with traditional optimization algorithms, game theory is another efficient way to solve resource allocation. The ordinary optimization algorithm is to find the optimal solution by solving the optimization expression, while the method of game theory is to find Nash equilibrium by iterations. Sometimes, there’s no optimal solution in the former one, or the solving will take much time. Thus, this paper adopts the later one, the game theory, to solve the uplink allocation problem in multi-UAV video transmission. Each UAV is regarded as a game player to compete with others.

Generally speaking, game methods can be divided into cooperative game and non-cooperative game. In practice, there is no absolute boundary between the two. From the perspective of a single player, each UAV intends to occupy the bandwidth as much as possible, but it is difficult to achieve due to the the limited total bandwidth the operator can provide. In addition, finally, the UCDC needs to improve the overall QoE of all videos. In this process, UAVs have a rational cooperation trend as well as a non-cooperative and selfish competition. Therefore, the Potential Game method can be used to build the model. On this basis, this paper believes that the behavior of game players is not completely independent, and the choice of game strategies depends on a kind of probability information outside the game process. Thus, the players can partially cooperate to change their strategies. Compared with the completely independent and non-cooperative Nash equilibrium, we hope to achieve the correlation equilibrium in this way and further improve the performance of the algorithm.

The game can be expressed as $G=[\Omega ,{\left\{K_{i}\right\}}_{i\in \Omega },{\left\{U_{i}\right\}}_{i\in \Omega }]$, where Ω={1,2,…,N} is the set of players, $K_{i}\in Cband$ represents the set of the strategies of the *i*-th player, and $U_{i}$ is the corresponding utility set. Thus, we have the pure strategy of player *i* as $r_{i}\in K_{i}$, and have $r_{-i}\in K_{1}\times \cdots \times K_{i-1}\times K_{i+1}\times \cdots \times K_{N}$ as the strategy of all the players except *i*, where × denotes Cartesian product. We hope that, by less information interaction, each player can choose a reasonable strategy without breaking the equilibrium. In this section, we construct a potential function as $I=\sum_{i=1}^{N}u_{i}$, and the problem can be presented as:

(6) $$\begin{array}{cc} \max\; & I=\Omega \cdot \sum_{i=1}^{N}\{a+b\cdot \sqrt{\frac{r_{i}}{c}}\left(1-\frac{c}{r_{i}}\right)\}-\eta \cdot \sum_{i=1}^{N}r_{i}+N\cdot \Lambda ,\\ s.t.\; & R_{i}^{\min}\leq r_{i}\leq R_{i}^{\max},\\ \; & 0\leq \sum_{i=1}^{N}r_{i}\leq Cband. \end{array}$$

**Theorem** **1.** *Although, as a participant in the game, each UAV player can change its video transmission rate strategy without cooperation, the whole game process takes the total utility of UCDC as the goal, which makes each player conduct rational cooperation. Therefore, this game process is a complete potential game.*

**Proof.**  For the *i*-th UAV, we assume that it has another rate strategy to choose, expressed as $r_{i}^{\prime }$. Because the potential function *I* is the sum of the single utilities, we can get □

(7) $$\begin{array}{cc} \Delta I & =I\left(r_{i}\right)-I\left(r_{i}^{\prime }\right)=u\left(r_{i}\right)-u\left(r_{i}^{\prime }\right)=\Delta u. \end{array}$$

The change of potential function is equal to the change of utility function of each player. According to [52], the game process *G* is a complete potential game, and the potential function *I* is a complete potential function. According to Theorem 1, in the process of rate allocation, as the participant of the game, a single UAV’s rate updating will change its own utility, while the changing value is just the same with the total utility’s variation. That means the application scenarios presented in this paper satisfies the definition of potential game. In order to maximize the total QoE utility, UAVs not only have to compete with each other, but also need to cooperate partially.

**Theorem** **2.** *In potential game $G=[\Omega ,{\left\{K_{i}\right\}}_{i\in \Omega },{\left\{U_{i}\right\}}_{i\in \Omega }]$, if the potential function can be expressed as $I=\Omega \cdot \sum_{i=1}^{N}\{a+b\cdot \sqrt{\frac{r_{i}}{c}}\left(1-\frac{c}{r_{i}}\right)\}-\eta \cdot \sum_{i=1}^{N}r_{i}+N\cdot \Lambda$, then the correlated equilibrium of the game exists and is unique.*

**Proof.**  From Equation (6), we can get the first-order partial derivative of the potential functionA □

(8) $$\begin{array}{c} \frac{\partial I}{\partial r_{i}}=\Omega \cdot (\frac{b}{2\sqrt{cr_{i}}}+\frac{b\sqrt{c}}{2{\sqrt{r_{i}}}^{3}})-\eta . \end{array}$$

Obviously, Equation (8) is a continuous function in the strategy space, so the potential function *I* is continuous and differentiable. Since $\Omega \cdot (\frac{b}{2\sqrt{cr_{i}}}+\frac{b\sqrt{c}}{2{\sqrt{r_{i}}}^{3}})$ and η>0, then there must be an extreme point $r_{i}={\tilde{r}}_{i}$ that can make Equation (8) identical to 0. Then, the second-order partial derivative of Equation (6) can be solved as:

(9) $$\begin{array}{c} {\left.\frac{\partial^{2}I}{\partial r_{i}^{2}}\right|}_{r_{i}={\tilde{r}}_{i}}=-\Omega \cdot \left[\frac{b}{4\sqrt{c}{\sqrt{{\tilde{r}}_{i}}}^{3}}+\frac{3b\sqrt{c}}{4{\sqrt{{\tilde{r}}_{i}}}^{5}}\right]. \end{array}$$

Because $\frac{b}{4\sqrt{c}{\sqrt{{\tilde{r}}_{i}}}^{3}}>0$ and $\frac{3b\sqrt{c}}{4{\sqrt{{\tilde{r}}_{i}}}^{5}}>0$, we can get ${\left.\frac{\partial^{2}I}{\partial r_{i}^{2}}\right|}_{r_{i}={\tilde{r}}_{i}}<0$.

The above results show that the potential function constructed in the potential game *G* is a continuously differentiable convex function in the strategy space. According to [53], potential game *G* has a correlation equilibrium, and the result is unique. In the process of rate allocation, UAVs compete for bandwidth as game players. When anyone’s single strategy change can no longer bring increase to the total utility function, the system reaches a stable and unique equilibrium state. At this point, the extreme rate strategy ${\tilde{r}}_{i}$ is the ultimate bandwidth choice for the *i*-th UAV. Thus, all the N UAVs will keep their own extreme rate strategies and the stability won’t be broken as long as the transmitting environment and the video characteristics remain unchanged.

## 5. Uplink Allocation Algorithm

The uplink allocation problem has been modeled as a potential game and we need to solve it for the correlated equilibrium. Generally, the process of solving Nash equilibrium is often a process of seeking the optimal solution by continuous iteration. Common iterative algorithms include Gauss–Seidel, Jacobi iterations, etc. The Jacobi iteration method is an earlier and simple algorithm, and its convergence speed is slow. Gauss–Seidel iteration is an improvement of the Jacobi algorithm. Although it has improved the convergence speed compared with the former one, it is still not suitable for real-time transmission service. In this paper, there is both competition and partial cooperation in the UAV cluster. In order to achieve a state of correlated equilibrium with a relatively fast convergence rate, the regret matching algorithm in [54] is adopted to design a distributed self-learning algorithm for the game model. The general idea of this algorithm is that the probability of a UAV changing its strategy is proportional to the regret degree of the UAV not choosing other strategies in the past. The implementation steps are as follows:

**(1) Initialization:**

Each player can choose the minimum rate as the first strategy from the strategy space {R_space} at the initial time t=1. Thus, we provide each UAV with a small initial video encoding rate at this step. In fact, the initial strategy can be any value in the rate range of the strategy space.

**(2) Iterative Update Process:**

*Strategy Update:*

When time t≥2, each player calculates the utility of the current strategy $r_{i}$ and the one of another strategy $r_{i}^{\prime }$, and gets the average difference between the two utilities:

(10) $$L_{i}^{t}\left(r_{i},r_{i}^{\prime }\right)=\frac{\lambda }{t}L_{i}^{\lambda }\left(r_{i},r_{i}^{\prime }\right)+\frac{1}{t}\left[u_{i}^{t}\left(r_{i}^{\prime },r_{-i}\right)-u_{i}^{t}\left(r_{i},r_{-i}\right)\right],$$

where λ denotes for the time before *t*, and that means λ<t. Then, $R_{i}^{t}\left(r_{i},r_{i}^{\prime }\right)=\max\left\{L_{i}^{t}\left(r_{i},r_{i}^{\prime }\right),0\right\}$ and it is a measure of “regretting”.

*Strategy Decision:*

Assuming that at time *t*, the *i*-th player chooses the strategy $r_{i}$, then at time t+1, the player will reconsider the strategy. The basis for its choice of strategy will follow the probability distribution:

(11) $$\left\{\begin{array}{c} \pi_{i}^{t+1}\left(r_{i}^{\prime }\right)=\frac{1}{\mu }R_{i}^{t}\left(r_{i},r_{i}^{\prime }\right)\;\;\forall r_{i}^{\prime }\neq r_{i},\\ \pi_{i}^{t+1}\left(r_{i}\right)=1-\sum_{r_{i}^{\prime }\neq r_{i}}\pi_{i}^{t+1}\left(r_{i}^{\prime }\right), \end{array}\right.$$

where μ>0, and it is big enough. According to this distribution, we can select the strategy with higher probability for player *i* in the strategy space {R_space}. After several iterations, the selected results will not be changed and the algorithm will converge. If each UAV follows the above distributed algorithm to update its strategy, then the whole potential game eventually converges to the correlated equilibria.

## 6. Simulation Results and Analyses

We conduct some simulations to evaluate the game model and the algorithm we proposed. Suppose, in the scenario shown in Figure 2, there are seven UAVs flying in a certain AP coverage. They shoot videos independently and send seven different videos back to UCDC via a wireless network. Their rate ranges and characteristics are marked in Table 1. Their motions can be divided into three categories: slow, medium and fast. The slower the motion is, the less transmission resources the video occupies. In addition, the rate is also related to the complexity of the scene. Complex scenarios will also take up more bandwidth. In a word, the seven videos here represent different typical shooting scenarios. In practice, the number of videos may be more, but the principle and workflow of the algorithm is the same.

### 6.1. Initial Analysis

**Figure 3:**
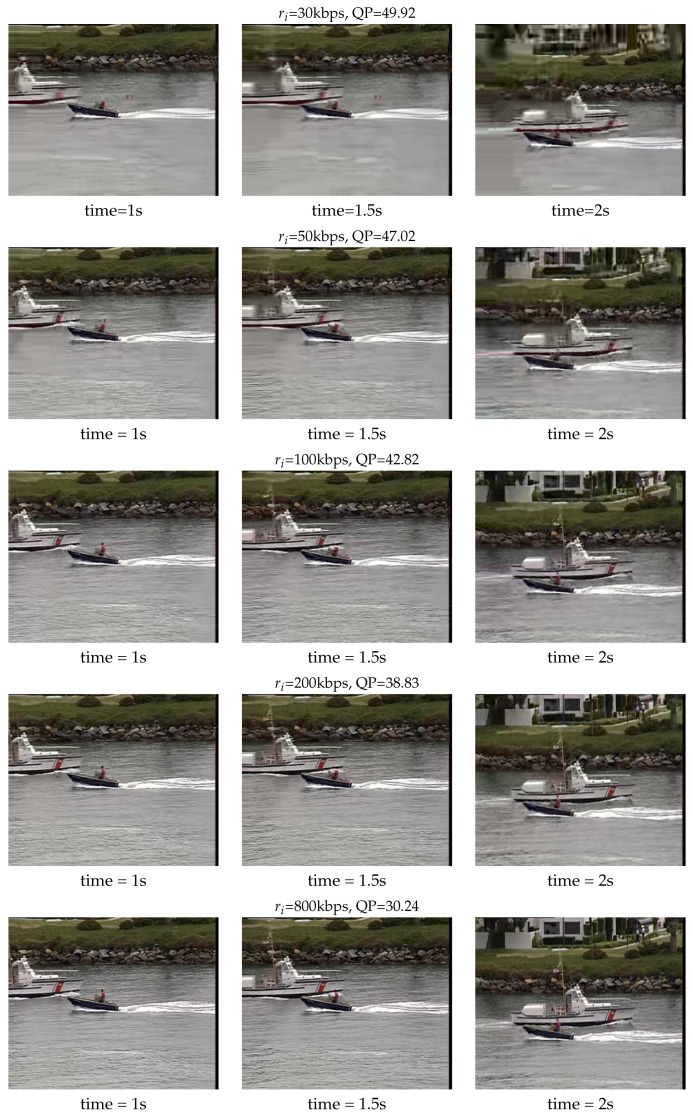
The frames of Coastguard after being encoded by H.265 in different rates.

When the same video is compressed and encoded in different rates, it will bring users different viewing experiences. In order to validate this effect, we first use FFmpeg codec to encode the CIF video Coastguard into H.265 files. The original video’s format is yuv420p, 352 × 288, and the frame rate is 30 fps. After the encoding, at the time 1 s, 1.5 s and 2 s, the compressed video frames are extracted for comparison, and the results are shown in Figure 3. The encoding rate $r_{i}$ is selected within the range shown in Table 1 and the corresponding Quantizer Parameter (QP) is also listed. When the encoding rate is low, the image is relatively fuzzy, the block effect is very obvious, and the experience quality is extremely bad. As the speed increases, the clarity is improved. When the rate is greater than 200 kbps, the effect of video is more easily accepted by users. Thus, if the UAV is assigned to a different bandwidth, the onboard encoder will update the encoding rate in order to achieve the new bandwidth, which will directly affect the video’s QoE. In the following simulation, we believe that each UAV’s encoder can achieve such adaptive video encoding. That means the rate variation of the encoder can synchronize with rate allocation. After the updating, the constant encoding rate equals to the newly specified bandwidth value in each iteration. We keep on observing the total utility changes brought by the new allocation, decide whether to change the rate, and further select the best allocation method according to the distributed self-learning algorithm.

The simulation is based on Matlab R2017a platform. Ref. [38] gave some QoS parameters of several typical wireless technologies which can be used in UAV communication. In addition, the data rates could vary from 50 kbps to 10 Gbps. Various wireless networks can provide different channel bandwidth. In order to make the simulation analysis more universal and convincing, we set the total channel bandwidth of the system according to the requirements of video transmission. We sum the minimum and maximum rates of seven videos in Table 1 and the results are $\sum_{i=1}^{7}R_{i}^{\min}$ = 622 kbps and $\sum_{i=1}^{7}R_{i}^{\max}$ = 5.485 Mbps. That means if the total bandwidth is less than 622 kbps, it cannot meet the minimum requirements and the transmission of one or more videos will fail. When the total bandwidth is more than 5.485 Mbps, it can always meet the maximum requirements of each video and each video could be sent at its maximum rates. Thus, resource allocation is no longer necessary in this condition. Therefore, we choose to change the total transmission bandwidth from 1 Mbps to 6 Mbps in the simulation. In other words, a certain value within this range can be assigned as Cband to verify the effect of the model and the algorithm we propose. In this way, the minimum transmission requirement of the seven videos can be guaranteed, but the maximum demand of each video cannot be met at the same time. Each UAV needs to acquire resources through competition. Therefore, the algorithm studied in this paper is mainly aimed at the situation of relatively insufficient resources. The users’ needs and the system’s ability of providing resources are always relative. Once the network cannot meet the highest requirements of all users, which leads them to compete for resources, we may adopt the allocation mechanism mentioned in this paper.

The encoding rate of each UAV plays a crucial role in this paper. It is both the cause and the result of strategy update. Thus, the rate range of each video in Table 1 is firstly segmented into some discrete values and all of them can form the strategy space {R_space} for each video. On one hand, different rate selection from the strategy space represents the application of different video encoding rates, which will lead to the change in the total utility function, in other words, in the potential function. On the other hand, the change of the potential function will drive the algorithm to update the allocation scheme. That means to change the bandwidth for each UAV. As a result, each encoders have to resort to a new encoding rate and the scheme will help to look for a new one from the strategy space {R_space} to replace the old—wherein, it follows the distributed self-learning algorithm mentioned above. Until the potential function is no longer changed, the system reaches equilibrium.

### 6.2. Convergence of the Algorithm

**Figure 4:**
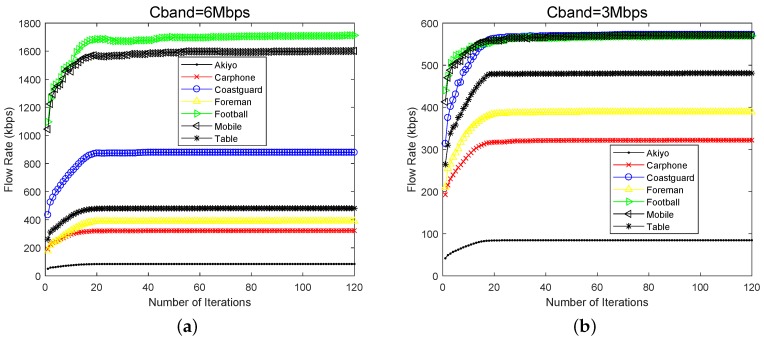

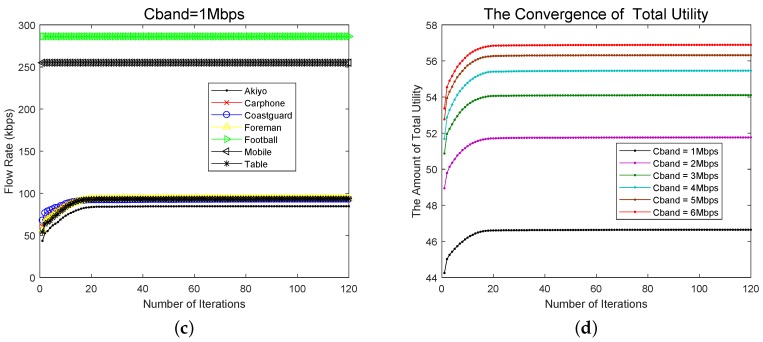
Number of iterations versus flow rate and total utility.

We first analyze the convergence of the algorithm. Figure 4 shows the real-time encoding rates of seven videos under three different total channel rates (1 Mbps, 3 Mbps and 6 Mbps). We can see that, after nearly 20 iterations, all curves become smooth and stable. This means that the proposed algorithm can make the system converge to equilibrium state in a very short time and obtain Nash equilibrium. When Cband = 6 Mbps, this means that the channel bandwidth is relatively adequate. As shown in Figure 4a, each video can be encoded and transmitted at its maximum rate $R_{i}^{\max}$, and faster and more complex video naturally takes up more resources. When Cband = 3 Mbps, the total bandwidth is limited. In Figure 4b, the videos, Mobile, Football and Costguard reduce their rates accordingly, thus keeping the total utility function within a reasonable range. When Cband = 1 Mbps, the overall channel environment is extremely poor. In Figure 4c, Football and Mobil can only maintain the minimum rate $R_{i}^{\min}$, and the rates of the rest videos are below 100 kbps. Next, we analyze the time-varying curve of the total utility value obtained by the whole system under different channel bandwidth conditions, as shown in Figure 4d. It can be seen that, after finite iterations, the total utility value also converges to the stable value. As long as the total bandwidth provided by the AP stays the same and the characteristics of each video remain unchanged, both the data rates of all UAVs and the total utility of the system will finally remain stable. The algorithm can converge rapidly under different bandwidth conditions and has very good stability.

### 6.3. Performance Analyses of Different Algorithms

To analyze the performance of the proposed algorithm, we compare it with some resource allocation algorithms in multi-rate and multi-user scenarios, including AFR, MSPSNR [55] and Average. AFR allocates the network bandwidth as fairly as possible to each user under the condition of satisfying the minimum and not exceeding the maximum video rate demand. Under the condition of meeting the minimum rate needs, MSPSNR gives bandwidth priority to the videos with slow or medium motion or a smooth scene, in order to improve the PSNR. The average allocates bandwidth equally without exceeding the maximum user requirement, although sometimes it cannot guarantee the minimum requirement.

**Figure 5:**
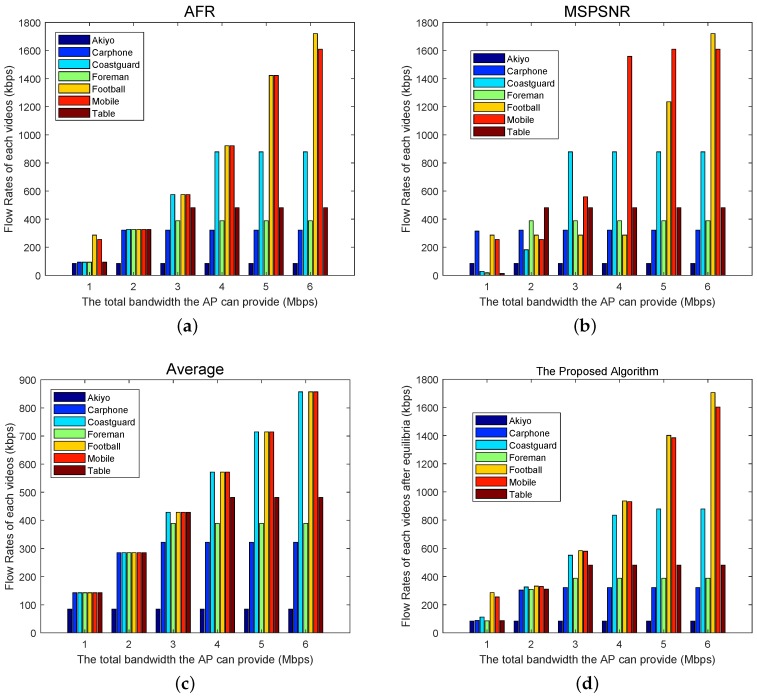
Allocated rates using different methods.

Figure 5 shows the rate allocation results of different methods under different bandwidth conditions. Figure 5a is the results of AFR. If the allocated bit-rate is within the rate range of each video, it will be equally divided. Otherwise, it will be allocated according to its maximum or minimum value. Figure 5b shows the results of MSPSNR. It first satisfies the minimum rate requirement of all videos. Then, if there is any remaining bandwidth, it is allocated to the video with the minimum $R_{i}^{\max}$ until its rate reaches this $R_{i}^{\max}$. This process continues until all bandwidth is allocated or all maximum rate requirements are satisfied. Figure 5c shows the result of the Average algorithm, which calculates the average bandwidth that could be allocated to each video. If the average bandwidth exceeds the maximum rate $R_{i}^{\max}$ of the video, it will be transmitted at $R_{i}^{\max}$. Otherwise, the average bandwidth value will be allocated to it. The implementation of this method is the simplest. Unfortunately, for some high-speed videos, the minimum requirements cannot always be met, and then the video will not be transmitted correctly. Figure 5d shows the allocation results of the proposed algorithm in this work. In order to maximize utility, each UAV iteratively updates the rate according to its own video characteristics and rate ranges. When the allocation algorithm converges, each user could be allocated reasonable bandwidth.

**Figure 6:**
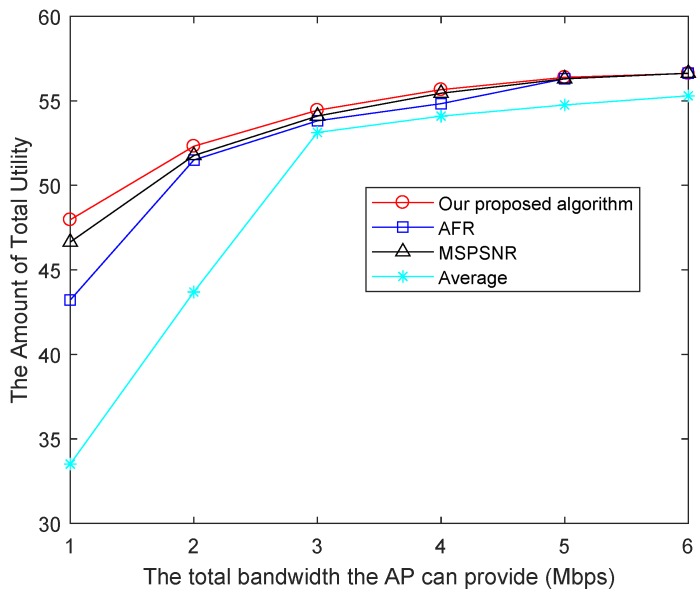
Total utility using different methods.

Figure 6 shows the total utility of different algorithms at different channel bandwidth. In the algorithm of this paper, a utility function of users’ QoE is designed and the problem is solved in order to maximize the utility. Thus, the total utility effects are better than other methods under different bandwidth conditions. The algorithm of Average has the worst performance because it does not consider the characteristics of videos. AFR takes the different needs of videos into account and stays fair. Its utility performance is much better than Average. Compared with AFR, the total utility of MSPSNR is properly improved.

### 6.4. Influence of the Cost Factor

**Figure 7:**
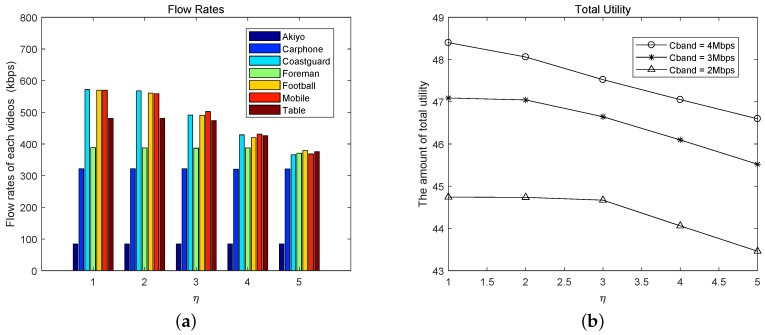
The influence of cost factor η.

From (5) in Section 3.2, we can find that the total loss in the utility function comes from the costs of both channel leasing and energy consumption. Thus, the factor η which combines the above two factors, θ and δ, can affect the results of rate allocation. When the total channel bandwidth provided by AP is 3 Mbps, we change the value of η to obtain the corresponding allocation results. From Figure 7a, we can find, when η becomes larger, the rates of videos with complex scene and fast motion will decrease most obviously, such as Coastguard, Football and Mobile. When η>4, Table’s rate also begins to decrease. When the total channel bandwidth is set to 2 Mbps, 3 Mbps and 4 Mbps, respectively, we can also find this corresponding reduction in each total utility curves from Figure 7b. Therefore, from the aspect of channel leasing, the NSP can effectively control the bandwidth allocation by adjusting the price parameter θ. This plays an important role in differentiating the service level of multimedia users and maintaining the robustness of the whole network. On the other hand, energy consumption should also be considered in video transmission of UAVs. It is related not only to the actual video transmission rate, but also to the distance between the UAV and AP. The energy factor δ in the utility function could to some extent describe the different energy consumption which is caused by different transmission distances. When the distance between the UAV and AP becomes obviously far, δ will increase and the transmission will consume more energy, which will affect the results of the resource allocation.

## 7. Conclusions

In this paper, the uplink channel allocation problem of multi-UAV video streaming is discussed. Not only the total video QoE, but also the cost for channel leasing and energy consumption is considered to formulate the utility function. Based on game theory, the distributed model is established, which further enhances the flexibility and robustness of the system. We have proved that the model converges to the correlation equilibria and have solved the model by the distributed self-learning algorithm. Simulation results show that the proposed mechanism can effectively solve the rate allocation problem of the UAV cluster.

## Figures and Tables

**Table 1:** Parameters of different videos.

	$R_{i}^{\min}\left(kbps\right)$	$R_{i}^{\max}\left(kbps\right)$	Style
Akiyo	1.5119	84.5447	slow motion and smooth scene
Carphone	20.2554	322.0153	medium motion and smooth scene
Table	12.7781	481.1014	medium motion and smooth scene
Foreman	17.8168	388.7091	medium motion and smooth scene
Coastguard	28.4987	878.8011	medium motion and complex scene
Football	286.311	1720	fast or complex motion
Mobile	225.0682	1610	fast or complex motion
